# P7C3 Ameliorates Bone Loss by Inhibiting Osteoclast Differentiation and Promoting Osteogenesis

**DOI:** 10.1002/jbm4.10811

**Published:** 2023-09-06

**Authors:** Bo Tian, Jinyu Bai, Lei Sheng, Hao Chen, Wenju Chang, Yue Zhang, Chenlu Yao, Chenmeng Zhou, Xiaoyu Wang, Huajian Shan, Qirong Dong, Chao Wang, Xiaozhong Zhou

**Affiliations:** ^1^ Department of Orthopedics The Second Affiliated Hospital of Soochow University Suzhou China; ^2^ Laboratory for Biomaterial and ImmunoEngineering, Institute of Functional Nano & SoftMaterials (FUNSOM) Soochow University Suzhou China

**Keywords:** BONE HOMEOSTASIS, OSTEOBLAST, OSTEOCLAST, OSTEOPOROSIS, P7C3

## Abstract

Bone homeostasis, the equilibrium between bone resorption and formation, is essential for maintaining healthy bone tissue in adult humans. Disruptions of this process can lead to pathological conditions such as osteoporosis. Dual‐targeted agents, capable of inhibiting excessive bone resorption and stimulating bone formation, are being explored as a promising strategy for developing new treatments to address osteoporosis. In this study, we investigated the effects of P7C3 on bone remodeling and its potential therapeutic role in osteoporosis treatment in mice. Specifically, P7C3 can remarkably suppress receptor activator of nuclear factor‐κB (NF‐κB) ligand (RANKL)‐induced osteoclast differentiation in bone marrow macrophages via the Akt‐NF‐κB‐NFATc1 signaling pathway. Additionally, RNA sequencing (RNAseq) analysis revealed that P7C3 promoted osteoblast differentiation and function through the Wnt/β‐catenin signaling pathway, thereby enhancing bone formation. Furthermore, μCT analysis and histological examination of bone tissues from P7C3‐treated mice showed attenuation of both Ti‐induced bone erosion and ovariectomy (OVX)‐induced bone loss. These findings suggest that P7C3 may have a novel function in bone remodeling and may be a promising therapeutic agent for the treatment of osteoporosis. © 2023 The Authors. *JBMR Plus* published by Wiley Periodicals LLC on behalf of American Society for Bone and Mineral Research.

## Graphical abstract

Maintaining bone homeostasis is crucial for healthy bone tissue in adults, and disruptions can cause osteoporosis. Dual‐targeted agents that inhibit excessive resorption and promote formation are being explored as new osteoporosis treatments. P7C3 was investigated on bone remodeling in mice and its potential therapeutic role in osteoporosis. P7C3 remarkably suppressed osteoclast differentiation through the Akt‐NF‐κB‐NFATc1 signaling pathway and promoted osteoblast differentiation through the Wnt/β‐catenin pathway, enhancing bone formation. P7C3 may have a promising therapeutic role in treating osteoporosis.

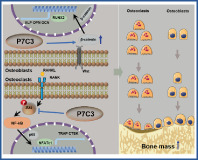

## Introduction

The skeleton is a dynamic tissue that serves as the body scaffold to protect vital organs, facilitating body movement and supporting hematopoiesis.^(^ ^1^ ^)^ Throughout the lifespan of organisms, the bones undergo constant remodeling through coordinated activities of osteoclasts and osteoblasts, which mediate bone resorption and formation, respectively.^(^ ^2^ ^)^ Maintaining a balance between resorption and formation is critical for preserving skeletal mass. However, with aging or certain diseases, bone homeostasis becomes disrupted, leading to an imbalance of the resorption over formation. This imbalance further contributes to bone loss and increased susceptibility to fragility fracture,^(^ ^2^, ^3^ ^)^ which affects hundreds of millions of people worldwide, especially postmenopausal women.^(^ ^4^ ^)^

Osteoclasts originate from the fusion of mononuclear macrophages lineage of hematopoietic stem cells, while osteoblasts derive from mesenchymal stem cells of bone marrow (BMSCs).^(^ ^5^ ^)^ However, these two types of cells are intertwined with each other. During the remodeling process, bone resorption occurs first, followed by bone formation in the resorption cavity. Osteoclasts not only play a bone‐resorbing role, but also secrete certain bone osteoblast‐stimulating factors, thereby regulating osteoblast activity.^(^ ^2^ ^)^ These certain factors promote the activation of the essential transcription factors runt‐related transcription factor 2 (Runx2), osterix (Osx), and β‐catenin,^(^ ^6^, ^7^, ^8^, ^9^, ^10^ ^)^ which govern the expression of osteoblast‐specific genes, including alkaline phosphatase (*ALP*), osteopontin (*OPN*), and osteocalcin (*OCN*),^(^ ^11^, ^12^ ^)^ thus exhibiting osteogenic function. Meanwhile, macrophage‐colony stimulating factor (M‐CSF) and the receptor activator of nuclear factor‐κB (NF‐κB) ligand (RANKL), produced by osteoblast‐lineage cells, play an essential role in osteoclast differentiation and function.^(^ ^13^, ^14^ ^)^ Binding between RANKL and its cell‐surface receptor RANK activates various downstream signaling pathways, including the mitogen‐activated protein kinase (MAPK: ERK, JNK, and p38), protein kinase B (Akt), and NF‐κB pathways.^(^ ^15^ ^)^ Subsequently, the activation of the key transcription factors c‐Fos and nuclear factor of activated T cells, cytoplasmic 1 (NFATc1), triggers the expression of specific genes associated with osteoclasts such as cathepsin K (*CTSK*), calcitonin receptor (*CTR*), tartrate‐resistant acid phosphatase (*TRAP*), matrix metalloprotein‐9 (*MMP‐9*), dendritic cell‐specific transmembrane protein (*DC‐STAMP*), and *V‐ATPase*,^(^ ^16^, ^17^ ^)^ thereby promoting the fusion of osteoclast precursors and exerting osteoclast activity. At the same time, osteoblasts also release osteoprotegerin (OPG), which acts as a decoy receptor of RANKL and negatively regulates osteoclast differentiation, affecting bone resorption function.

Pharmacological treatments for osteoporosis that target bone remodeling can be classified into two main categories: antiresorptive drugs and anabolic drugs.^(^ ^18^ ^)^ These interventions or treatments can indeed affect both osteoclasts and osteoblasts simultaneously. However, anti‐resorptive therapies, such as denosumab (DMAb) and bisphosphonates, are often associated with a decrease in osteoblast activity, which leads to reduced bone formation and limits their effectiveness.^(^ ^2^, ^19^ ^)^ The gastrointestinal side effects and atypical femoral fractures associated with bisphosphonates have also raised concerns among patients and physicians.^(^ ^4^, ^20^ ^)^ The other class is parathyroid hormone (PTH) and its analog. However, at higher doses or for prolonged periods, PTH can also stimulate osteoclastic activity, leading to bone resorption. Moreover, PTH treatment can trigger the release of calcium from bone, increasing the risk of hypercalcemia.^(^ ^21^ ^)^ In addition to the above mentioned mainstream clinical anti‐osteoporosis drugs, romosozumab provides a promising new direction for the dual regulation of bone remodeling by promoting bone formation and inhibiting bone resorption simultaneously. Regrettably, its cardiovascular risks have led to the US Food and Drug Administration's (FDA's) rejection of its initial application.^(^ ^4^ ^)^ Thus, developing new dual target–directed drugs that modulate bone remodeling with limited side effects remains a promising strategy for the treatment of osteoporosis.

P7C3, an aminopropyl carbazole compound that was discovered through a target‐agnostic screening approach, exerts remarkable neuroprotective efficacy with few adverse effects^(^ ^22^, ^23^ ^)^ in several animal models of neurodegenerative diseases^(^ ^23^, ^24^, ^25^, ^26^, ^27^ ^)^ or nerve cell injury.^(^ ^28^, ^29^, ^30^, ^31^, ^32^ ^)^ Additionally, research has shown that P7C3 may have a protective effect against liver and spinal cord injuries.^(^ ^33^, ^34^, ^35^ ^)^ However, the effect of P7C3 on bone remodeling has not been reported. In this study, we revealed that P7C3 exhibited a property to serve as a bone remodeling dual regulator and attenuate bone loss in various osteoporosis mouse models. The results showed that P7C3 inhibits osteoclast differentiation and affects bone resorption by targeting specific molecular pathways. Moreover, P7C3 promotes osteoblast differentiation and bone formation. These findings establish the mechanisms underlying the activity of P7C3 in bone remodeling and provide a basis for further pharmacological studies of this compound.

## Materials and Methods

### Study approval

All animal and human experimental procedures followed were reviewed and approved by the Ethics Committee of Soochow University (Approval No. SUDA20220701A02).

### Mice and reagents

C57BL/6 mice (female, 6–8 weeks old) were purchased from the Laboratory Animal Center of Soochow University. During the experimental period, the mice were housed in ventilated cages in a specific pathogen‐free facility at room temperature (22–24°C) and kept on a 12‐h light/dark cycle, with water *ad libitum* and standard chow. P7C3 was obtained from MedChemExpress (Monmouth Junction, NJ, USA) and stored in dimethyl sulfoxide (DMSO) at −20°C. Recombinant mouse macrophage‐colony stimulating factor (M‐CSF) and RANKL were purchased from R&D Systems (Minneapolis, MN, USA). Soluble, recombinant human M‐CSF, RANKL and tumor growth factor β (TGF‐β) were obtained from PeproTech (London, UK). Anti‐Akt, anti‐phospho‐Akt, anti‐ERK, anti‐phospho‐ERK, anti‐p38, anti‐phospho‐p38, anti‐JNK, anti‐phospho‐JNK, anti‐NFATc1, anti‐CTSK, anti‐MMP‐9, and anti‐GAPDH antibodies were purchased from Cell Signaling Technology (Beverly, MA, USA). Anti‐TRAF‐6, anti‐IκB, anti‐p65‐NF‐κB, and anti‐phospho‐p65‐NF‐κB antibodies were purchased from Abcam (Cambridge, MA, USA). Alpha‐minimum essential medium (α‐MEM), fetal bovine serum (FBS), streptomycin, and penicillin were obtained from Gibco BRL (Grand Island, NY, USA).

### Cell proliferation and viability

The cytotoxicity of P7C3 on precursor cells of the osteoclastic and osteogenic lineages was determined with 3‐(4,5‐dimethylthiazol‐2yl)‐2,5‐diphenyltetrazolium bromide (MTT) assay. Bone marrow–derived macrophages (BMMs), RAW264.7, bone mesenchymal stem cells (BMSCs), and MC3T3‐E1 cells were seeded in 96‐well plates and proliferated in full ɑ‐MEM medium (α‐MEM containing 10% FBS, 1% penicillin and streptomycin) at a density of 5 × 10^3^ cells/well. With the indicated concentrations of P7C3 added for 1or 2 days, the medium containing MTT (5 mg/mL) was replaced in each well and incubated for an additional 2 h at 37°C. Then, the crystals were dissolved in DMSO and detected at 490 nm (OD490) by a Bio‐Tek microplate reader (BioTek, Winooski, VT, USA). Compared with the absorbance values of untreated controls, cell viability was calculated.

### In vitro osteoclastogenesis assay

Six to eight‐week‐old female C57BL/6J mice were euthanized to prepare BMMs as described.^(^ ^36^ ^)^ Briefly, bone marrow cells (BMCs) were obtained by flushing the marrow cavities of the femur and tibia with α‐MEM and then cultured in full α‐MEM medium (α‐MEM containing 10% FBS, 1% penicillin and streptomycin) at 37°C/5% CO_2_ overnight. Nonadherent cells were harvested and further cultured in complete α‐MEM containing 30 ng/mL M‐CSF (R&D Systems) for 3 days to generate BMMs. To generate osteoclasts, BMMs were plated at a density of 1 × 10^4^ cells/well in a 96‐well plate in full medium containing 30 ng/mL M‐CSF and 50 ng/mL RANKL (R&D Systems) for 5–7 days with the indicated concentrations of P7C3. After the cells were fixed with 4% paraformaldehyde for 20 minutes, an acid phosphatase leukocyte kit (Sigma‐Aldrich, Darmstadt, Germany) was used for TRAP staining. TRAP^+^ multinucleated cells with three or more nuclei were counted as osteoclasts under an Olympus (Waltham, MA, USA) microscope.

For human osteoclast differentiation, peripheral blood mononuclear cells (PBMCs) of normal healthy authors were obtained from ethylenediamine tetraacetic acid (EDTA)‐blood using Ficoll (Lymphoflot; Bio‐Rad Laboratories, Hercules, CA, USA) density gradient centrifugation. PBMCs were cultured in a complete medium containing 30 ng/mL of M‐CSF, 3 ng/mL of RANKL, and 1 ng/mL TGF‐β (all PeproTech, Rocky Hill, NJ, USA) with the indicated concentrations of P7C3 for 7 days to generate osteoclasts.

### In vitro bone resorption assay

BMMs were cultured on 24‐well plates coated with hydroxyapatite (Corning Inc., Corning, NY, USA) in full medium containing 30 ng/mL M‐CSF and 50 ng/mL RANKL with the indicated concentrations of P7C3. The medium was changed every 2 days. After 5 days, the cells were cleaned with 5% sodium hypochlorite and the resorption pits were photographed by a microscope. The bone resorption area was analyzed and quantified using the software ImageJ (NIH, Bethesda, MD, USA; https://imagej.nih.gov/ij/).

### NF‐κB activation and nuclear translocation assay

RAW264.7 cells were cultured with complete medium, 50 ng/mL RANKL or 50 ng/mL RANKL plus 10μM P7C3 for 24 hours. Fixing fluid was added to the cells after the culture medium was removed. Then, the cells were washed three times with washing solution and blocked for 1 hour in blocking buffer. Following overnight incubation with the primary antibody at 4°C, cells were incubated with a secondary antibody conjugated to Cy3 for 1 hour at room temperature. 4′,6‐diamidino‐2‐phenylindole (DAPI) was added to stain nuclei for 5 minutes and then washed three times. After that, confocal microscopy was used to visualize the samples. All steps above were carried out according to the instructions contained in the NF‐κB activation and nuclear translocation assay kit (Beyotime Biotech, Shanghai, China).

### In vitro osteoblast differentiation and mineralization

BMSCs were isolated from C57BL/6 mice (female, 6–8 weeks old) as in our previous works.^(^ ^37^, ^38^ ^)^ The cells were cultured in 12‐well plates in osteogenic medium (complete DMEM, 10mM β‐glycerophosphate, 50 μg/mL ascorbic acid, and 10nM dexamethasone) with or without P7C3. For alkaline phosphatase (ALP) staining, a BCIP/NBT Alkaline Phosphatase Color Development Kit (Beyotime Biotech, Shanghai, China) was used to stain cells following osteogenic induction for 14 days. For Alizarin red staining (ARS), the cells were stained with Calcium Stain Kit (Modified Alizarin Red S Method) (Solarbio, Beijing, China) after 21 days of osteogenic differentiation. The ALP and ARS staining regions were then visualized under a microscope and measured using the software ImageJ.

### Quantitative real‐time PCR (qPCR)

Total RNA was extracted with TRIzol reagent (Biosharp, Hefei, China) according to the manufacturer's instructions. The cDNA was obtained by reverse transcription of total RNA using NovoScript®Plus All‐in‐one 1st Strand cDNA Synthesis SuperMix (gDNA Purge). Then, the qRT‐PCR product was amplified with the NovoStart® SYBR Quantitative real‐time PCR (qPCR) SuperMix Plus (Novoprotein, Shanghai, China). The expression of target genes was normalized to that of glyceraldehyde‐3‐phosphate dehydrogenase (GAPDH). A list of specific primer sequences is provided in Table S1.

### Western blot analyses

To extract the protein, the cells were washed with phosphate‐buffered saline (PBS) and lysed on ice in a radioimmunoprecipitation assay (RIPA) buffer containing protease inhibitors. The supernatants were then obtained by centrifugation at 12,000*g* for 15 minutes and quantified with a bicinchoninic acid (BCA) protein assay kit. An equal amount of protein was separated using sodium dodecyl sulfate–polyacrylamide gel electrophoresis (SDS/PAGE) gels and transferred onto polyvinylidene difluoride (PVDF) membranes (Bio‐Rad, Hercules, CA, USA). With 5% nonfat milk, the membranes were blocked and incubated with relevant primary antibodies at 4°C overnight. The next day, the membranes were incubated with appropriate horseradish peroxidase (HRP)‐conjugated secondary antibodies and visualized with chemiluminescent substrate (ECL). The gray bands of the proteins were quantized using the software ImageJ. The original data of Western blots is provided in Fig. S4.

### RNA sequencing

MC3T3‐E1 cells were cultured in six‐well plates in osteogenic medium for 3 days in the presence or absence of P7C3. Total RNA from the harvested cells was extracted with TRIzol reagent and assessed by Agilent Bioanalyzer (Agilent, Waldbronn, Germany). RNA libraries were produced using NEB Next® Ultra II RNA Library Prep Kit for Illumina and then sequenced on an Illumina HiSeq 4000 platform (Illumina, San Diego, CA, USA). Low‐quality reads and adapter sequences were removed from raw reads. Then, reads were aligned against the mouse genome with v.GRCm38.91. Based on this matrix, differential genes expressed between two groups were identified (R v3.5.1, DESeq2 v1.20). A gene expression that changes more than 1.3‐fold and *p* < 0.05 was regarded as significantly different. The genes obtained were further analyzed, for instance through Kyoto Encyclopedia of Genes and Genomes (KEGG) enrichment analysis.

### Animal model

Female C57BL/6 mice, 8 weeks old, were divided randomly and evenly into three groups: sham, Ti, and Ti plus P7C3. With the exception of the sham group, 30 mg Ti particles were embedded beneath the periosteum, at the midpoint of the sutural line. To evaluate the therapeutic effect of P7C3 on Ti particle‐induced bone destruction, the mice were treated with vehicle (sham and Ti group) or P7C3 (20 mg/kg in Ti plus P7C3 group) every day through intraperitoneal injection. After 14 days, the calvariae were collected for further μCT and histological analysis.

To study the effects of P7C3 on ovariectomy (OVX)‐induced osteoporosis model, 8‐week‐old female C57BL/6 mice were randomly divided into three groups: sham, OVX, and OVX plus P7C3. With the exception of the Sham group, the female mice were bilaterally ovariectomized. The uterus weight and weight development were assessed to confirm successful OVX. Six weeks after surgery, P7C3 (20 mg/kg) was intraperitoneally administered to the OVX mice once a day. Two weeks later, serum and bone tissue samples were collected for subsequent experiments.

### μCT analysis and bone histomorphometry

μCT analysis (SkyScan 1174; SkyScan, Aartselaar, Belgium) was performed with the consistent parameters (voxel size, 10.3 μm; X‐ray voltage, 50 kV; electric current, 810 μA; rotation step, 0.5 degrees). The three‐dimensional (3D) models of the calvaria and distal femur were reconstructed using the analysis program CTvol software. For quantitative assessment, static parameters such as bone mineral density (BMD), bone volume/tissue volume (BV/TV %), porosity percentage (%), bone surface/tissue volume (BS/TV), trabecular separation (Tb. Sp), and trabecular bone number (Tb.N) of each sample were measured.

Following μCT analysis, the bones were decalcified for sectioning and staining. To evaluate the bone trabecular structure and identify osteoclasts, 5‐μm‐thick sections were stained with hematoxylin and eosin (H&E) or TRAP. The parameters of bone tissues were quantified with the OsteoMeasure software (OsteoMetrics Inc., Decatur, GA, USA). To analyze the bone segment with immunofluorescence, 8‐μm‐thick longitudinally oriented sections were stained with anti‐Osterix (Abcam; ab209484). The relative expression levels were quantized by ImageJ.

### Blood biochemicals analysis

Blood samples were allowed to clot for 30 minutes and centrifuged at 3500*g* for 10 minutes. Aliquots of serum were prepared and stored at −80°C. Enzyme‐linked immunosorbent (ELISA) was employed to measure mouse serum RANKL and OPG according to the manufacturer's protocols (R&D Systems).

### Cytotoxicity evaluation in vivo

To evaluate the toxicity of the treatment, the main organs of treated mice were separated for H&E staining. Additionally, serum concentrations of aspartate aminotransferase (AST), alanine aminotransferase (ALT), blood urea nitrogen (BUN), and creatinine were determined.

### Statistical analysis

A minimum of three experiments were conducted, with quantitative data presented as the mean ± standard deviation (SD). The statistical analysis was conducted using the GraphPad Prism 8 software (GraphPad Software, Inc., La Jolla, CA, USA) with one‐way analysis of variance (ANOVA) or Student two‐tailed *t* tests. In the graphs, statistical significance is shown as *p < 0.05, **p < 0.01, ***p < 0.001, ****p < 0.0001. No significance is indicated by “ns.”

## Results

### P7C3 inhibited RANKL‐induced osteoclast differentiation and bone resorption

**Fig. 1:**
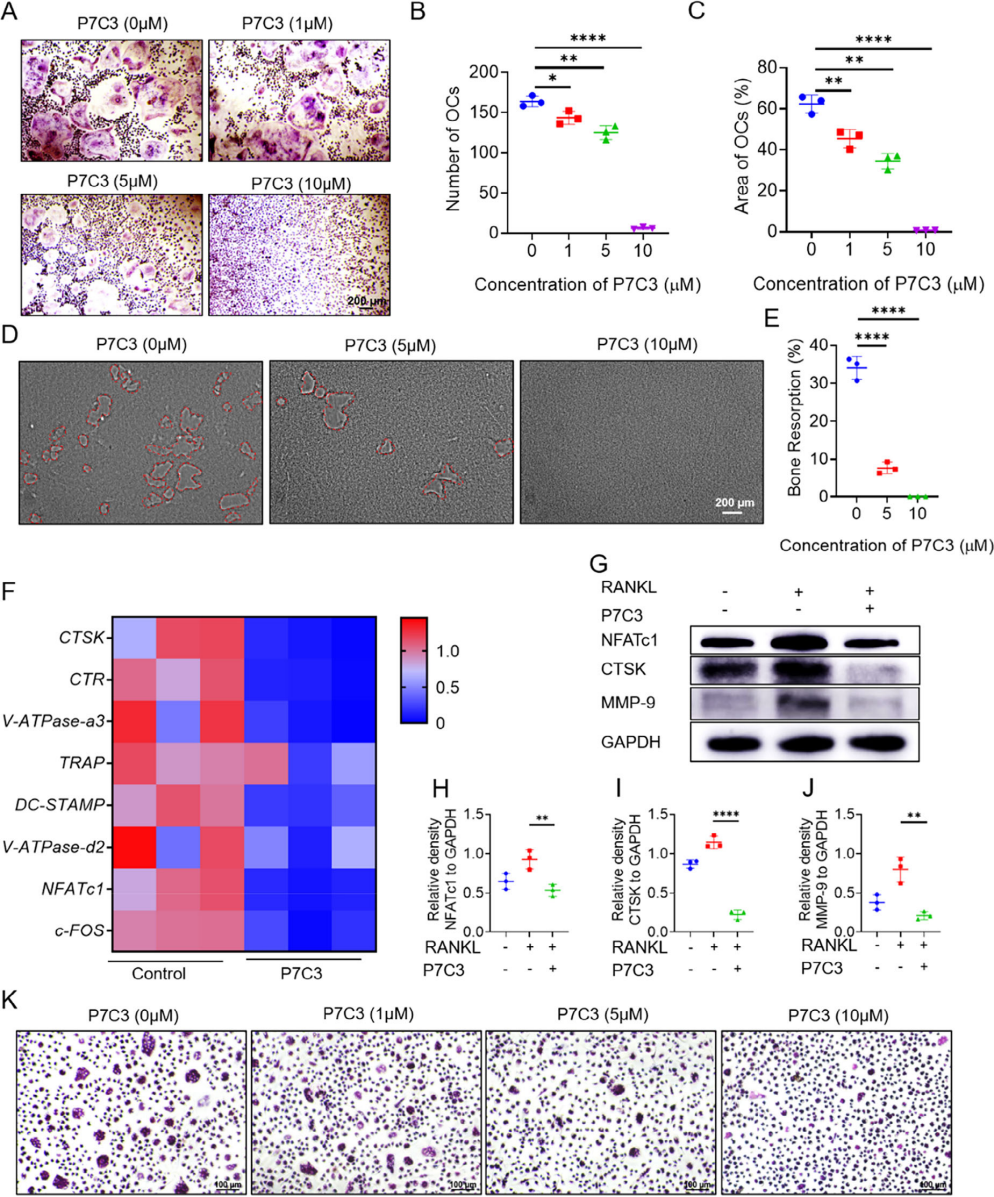
P7C3 inhibits osteoclast differentiation and bone resorption in vitro. (*A*) TRAP staining of osteoclasts derived from bone marrow cells treated with various concentrations of P7C3. (*B*) Quantification of the number of osteoclasts in *A*. (*C*) Quantification of the TRAP‐positive area in *A*. (*D*) Bone resorption assay of osteoclasts induced with different concentrations of P7C3. (*E*) Quantification of the area of bone resorption in *D*. (*F*) mRNA expression of osteoclast‐specific genes with and without P7C3 treatment, as measured by qPCR. (*G*) Western blot analysis of osteoclast‐specific proteins with and without P7C3 treatment. (*H*–*J*) Relative quantification of the proteins in *G*. (*K*) TRAP staining of osteoclasts derived from peripheral blood mononuclear cells treated with various concentrations of P7C3. Data are presented as mean ± SD; Statistical significance was calculated by one‐way ANOVA. *p < 0.05, **p < 0.01, ***p < 0.001. *n* = 3.

P7C3 is a small‐molecular compound with neuroprotective effects. To test whether it can also modulate bone metabolism, we investigated its effects on osteoclast differentiation in vitro. As assessed by TRAP staining and bone resorption pit assay, P7C3 treatment led to a significant decrease in the number and size of osteoclasts in a concentration‐dependent manner (Fig. 1*A*–*E*). With the concentration at 10μM, P7C3 significantly reduced the number of osteoclasts and the area of bone resorption pits, compared to the control group (vehicle‐treated cells). Notably, P7C3 did not affect cell survival or proliferation (Fig. S1A,B), indicating that its effect on osteoclast differentiation was not due to cytotoxicity or growth inhibition.

The expression levels of osteoclast‐specific genes and proteins further confirmed the inhibitory effect of P7C3 on osteoclast differentiation. As shown in Fig. 1*F*, P7C3 treatment significantly reduced the mRNA expression levels of osteoclast‐specific genes, including *CTSK*, *CTR*, *V‐ATPase*, *TRAP*, *DC‐STAMP*, *NFATc1*, and *c‐FOS*, 3 days after co‐incubation with the osteoclastogenic factor RANKL. Consistently, P7C3 treatment also decreased the protein expression levels of NFATc1, CTSK, and MMP‐9 (Fig. 1*G*–*J*). These results suggest that P7C3 may interfere with the early differentiation process of osteoclasts, leading to a decrease in their fusion and activity.

We further evaluated the effect of P7C3 on osteoclast differentiation induced by human peripheral blood cells. As shown in Fig. 1*K* and Fig. S2A, P7C3 also inhibited the differentiation of osteoclasts in a concentration‐dependent manner. Intriguingly, the inhibitory effect of P7C3 on human osteoclast differentiation was more pronounced than that on mice, indicated by the significantly lower number and size of human osteoclasts. These suggest that P7C3 may have a potential translational value in the treatment of bone diseases in humans.

### P7C3 suppressed RANKL‐induced Akt‐NF‐κB‐NFATc1 signaling

**Fig. 2:**
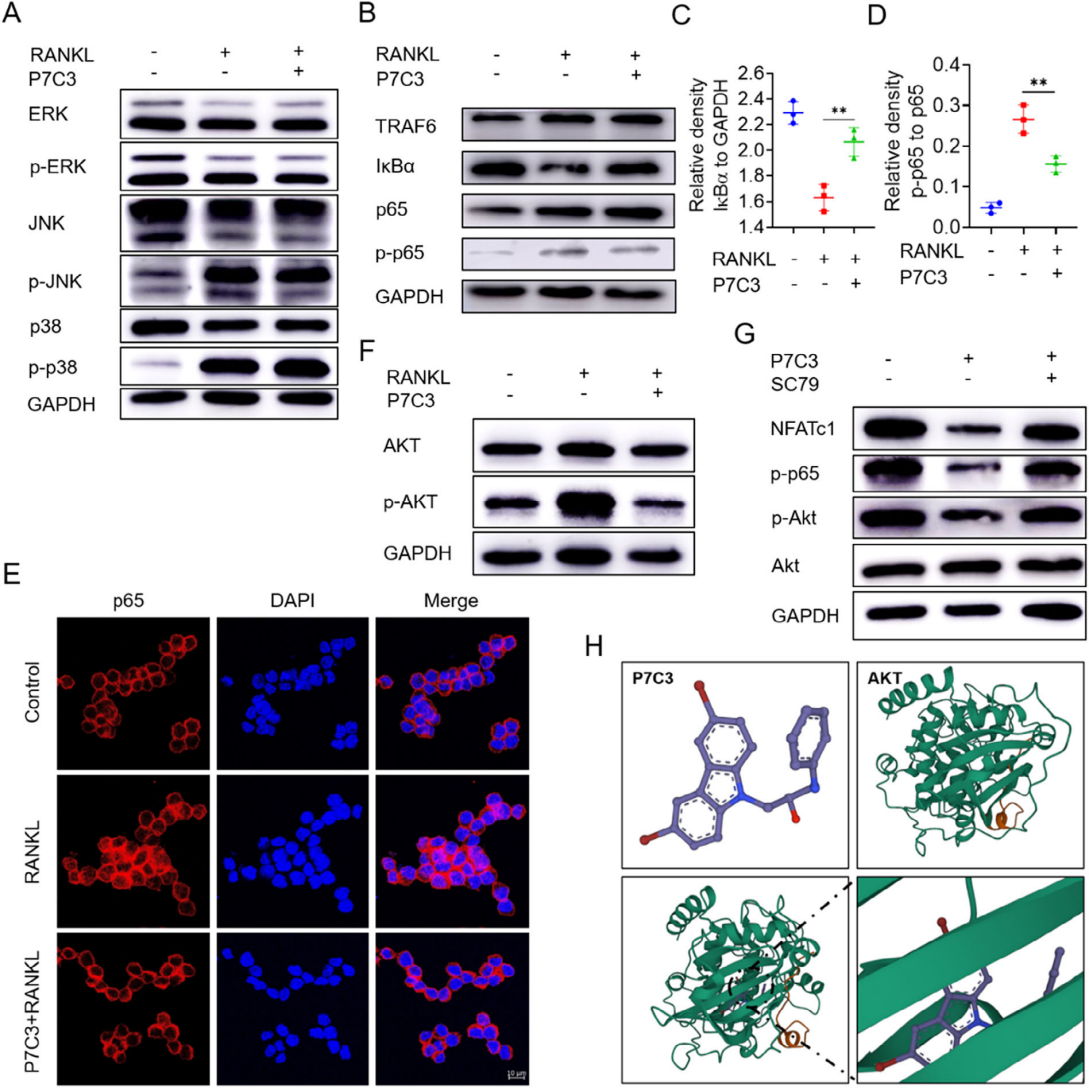
P7C3 inhibits osteoclast differentiation via the Akt‐NF‐κB‐NFATc1 pathway. (*A*) Effects of P7C3 (10μM) on MAPK signaling pathways (ERK, JNK, p38) in osteoclast differentiation. (*B*) Effects of P7C3 (10μM) on NF‐κB pathway in osteoclast differentiation. (*C*,*D*) Relative quantitative analysis of proteins involved in the NF‐κB pathway in *B*. (*E*) Immunofluorescence staining of p65 nuclear translocation in RAW264.7 cells induced with or without P7C3 (10μM) during osteoclast differentiation. (*F*) Effects of P7C3 (10μM) on Akt phosphorylation during osteoclast differentiation, as measured by Western blot analysis. (*G*) Effects of Akt phosphorylation activator SC79 on downstream NF‐κB pathway activation inhibited by P7C3 (10μM). (*H*) Docking analysis of small molecule P7C3 with Akt protein, showing binding energy of −9.328 kcal/mol. Data are presented as mean ± SD; statistical significance was calculated by one‐way ANOVA. *p < 0.05, **p < 0.01. *n* = 3.

To investigate the mechanism underlying the inhibitory effect of P7C3 on osteoclast differentiation, we performed Western blot analysis to examine the key signaling proteins involved in RANKL‐induced osteoclastogenesis. We found that treatment of P7C3 did not affect the expression levels of MAPK pathway proteins (Erk, JNK, p38) (Fig. 2*A* and Fig. S2B–D). However, the protein levels of p65, a key component of the NF‐kB signaling pathway, were significantly decreased after P7C3 treatment (Fig. 2*B*–*D*). Moreover, P7C3 treatment was associated with a decrease in the phosphorylation level of p65 and its nuclear translocation, consistent with the inhibitory effect of P7C3 on osteoclastogenesis (Fig. 2*E*).

In order to investigate whether the NF‐κB pathway is indirectly targeted by P7C3, we examined the expression levels of Akt, a known upstream regulator of NF‐κB signaling in P7C3‐treated cells. P7C3 treatment decreased the phosphorylation level of Akt (Fig. 2*F* and Fig. S2E), suggesting that P7C3 may regulate NF‐κB signaling in an Akt‐dependent manner. To validate this hypothesis, we pretreated the cells with a phospho‐Akt activator, SC79, which could activate Akt and promote its downstream signaling. As expected, SC79 treatment rescued the inhibitory effect of P7C3 on the phosphorylation level of p65 and the expression level of NFATc1, which is a key transcription factor involved in osteoclast differentiation (Fig. 2*G* and Fig. S2F–H). These results indicate that the Akt‐NF‐kB‐NFATc1 signaling pathway is involved in the inhibitory effect of P7C3 on osteoclast differentiation.

Next, we performed molecular docking simulations using AutoDock software to further explore the molecular basis of the interaction between P7C3 and Akt protein. The results showed that P7C3 can form hydrogen bonds with important residues in the active site of Akt, potentially stabilizing the Akt‐P7C3 complex (Fig. 2*H*). These findings support the hypothesis that P7C3 may directly interacts with Akt and modulates its activity and downstream signaling. Overall, we indicate that P7C3 inhibits osteoclast differentiation by targeting the Akt‐NF‐kB‐NFATc1 pathway, likely through direct interaction with Akt.

### P7C3 stimulated osteoblast differentiation

**Fig. 3:**
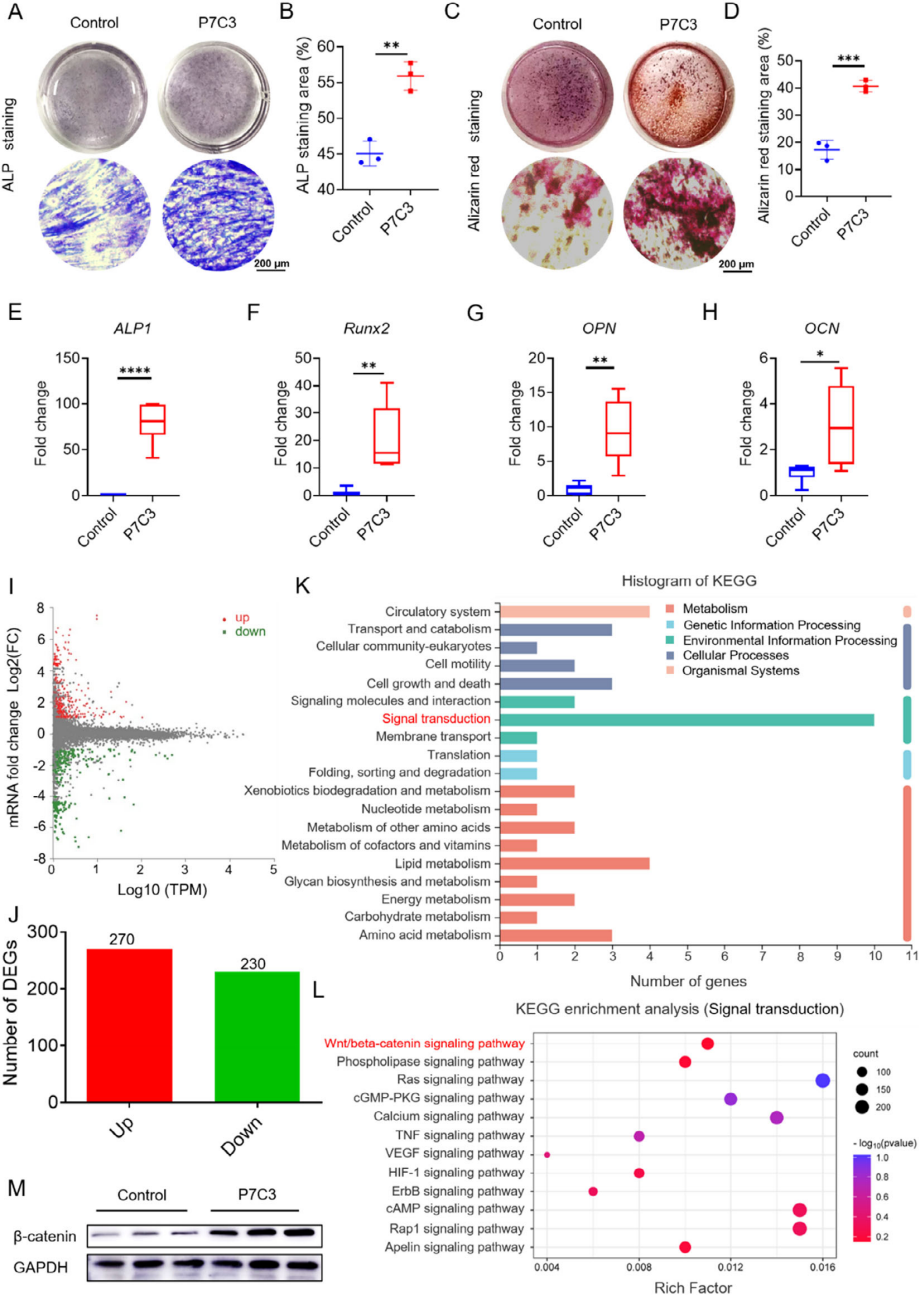
P7C3 promotes osteoblast differentiation and mineralization via the Wnt/β‐catenin pathway. (*A*) ALP staining of osteoblasts induced with or without P7C3 (10μM) during osteogenic differentiation. (*B*) Relative quantification of ALP staining area in *A*. (*C*) Alizarin red staining of mineralized nodules formed in osteoblasts induced with or without P7C3 (10μM) during osteogenic differentiation. (*D*) Relative quantification of Alizarin red staining area in *C*. (*E*–*H*) mRNA expression of osteoblast‐specific genes with and without P7C3 (10μM) treatment, measured by qPCR. (*I*) Volcano plot of differentially expressed genes identified by RNA sequencing analysis of osteoblasts induced with or without P7C3 during osteogenic differentiation. (*J*) The number of upregulated and downregulated genes identified by RNA sequencing analysis in *I*. (*K*) KEGG pathway enrichment analysis of differentially expressed genes in *I*. (*L*) Bubble plot showing specific signaling pathways identified by KEGG enrichment analysis in *K*. (*M*) Western blot analysis of β‐catenin expression in osteoblasts induced with or without P7C3 (10μM) during osteogenic differentiation. Data are presented as mean ± SD; statistical significance was calculated by Student *t* test. *p < 0.05, **p < 0.01, ***p < 0.001, ****p < 0.0001. *n* = 3.

Osteoblasts and osteoclasts work together to ensure proper bone remodeling. In the previous sections, we verified P7C3 is capable of inhibiting osteoclast differentiation and bone resorption. We further investigated its effect on osteoblast differentiation and function to explore the effect of P7C3 on bone remodeling. First, we confirmed that P7C3 was not cytotoxic to osteoblasts and showed good biocompatibility within the used concentration range (Fig. S1C,D). Then, we investigated the effect of P7C3 on osteoblast differentiation and function. As shown in Fig. 3*A*–*D*, P7C3 treatment triggered a significant increase in ALP activity and mineralized nodule formation, suggesting that P7C3 promotes osteoblast differentiation and mineralization. Moreover, we analyzed the expression levels of several osteoblast‐specific genes and markers by real‐time PCR analysis to confirm this observation at the molecular level. As shown in Fig. 3*E*,*F*, at day 3 after the induction of osteogenic differentiation, P7C3 treatment has already resulted in a significant upregulation of the expression of early osteoblasts differentiation markers, such as *ALP* and *RUNX2*. Besides, the expression levels of late markers of osteoblast differentiation including *OPN* and *OCN* (Fig. 3*G*,*H*), were significantly elevated at day 7 after osteogenic induction, indicating that P7C3 may enhance both early‐ and late‐stage osteoblast differentiation. These results demonstrate that P7C3 can promote osteoblast differentiation and maturation in vitro.

To further explore the mechanisms underlying the effects of P7C3 on osteoblast differentiation, we used RNA sequencing to examine the transcriptomic changes in osteoblast precursor cells treated with P7C3. We found that P7C3 treatment induced significant changes of 500 genes expression, including 270 upregulated and 230 downregulated genes (Fig. 3*I*,*J*). KEGG pathway enrichment analysis revealed that the differentially expressed genes were highly enriched in signaling pathways (Fig. 3*K*). Further pathway enrichment analysis focusing on bone‐related functions showed that the Wnt/β‐catenin signaling pathway was significantly upregulated by P7C3 treatment (Fig. 3*L*). To validate the upregulation of the Wnt/β‐catenin signaling pathway by P7C3, we examined the protein level of β‐catenin, the central component of the pathway. As shown in Fig. 3*M* and Fig. S2I, P7C3 treatment resulted in a significant increase in the protein level of β‐catenin, indicating P7C3 could activate the Wnt/β‐catenin signaling pathway. Taken together, we have demonstrated that P7C3 could promote osteoblast differentiation and mineralization without cytotoxicity. The transcriptomic changes induced by P7C3 suggest that its promotion effect on osteoblast differentiation might be mediated by the activation of the Wnt/β‐catenin signaling pathway.

### P7C3 suppressed Ti particle–induced calvarial osteolysis in vivo

**Fig. 4:**
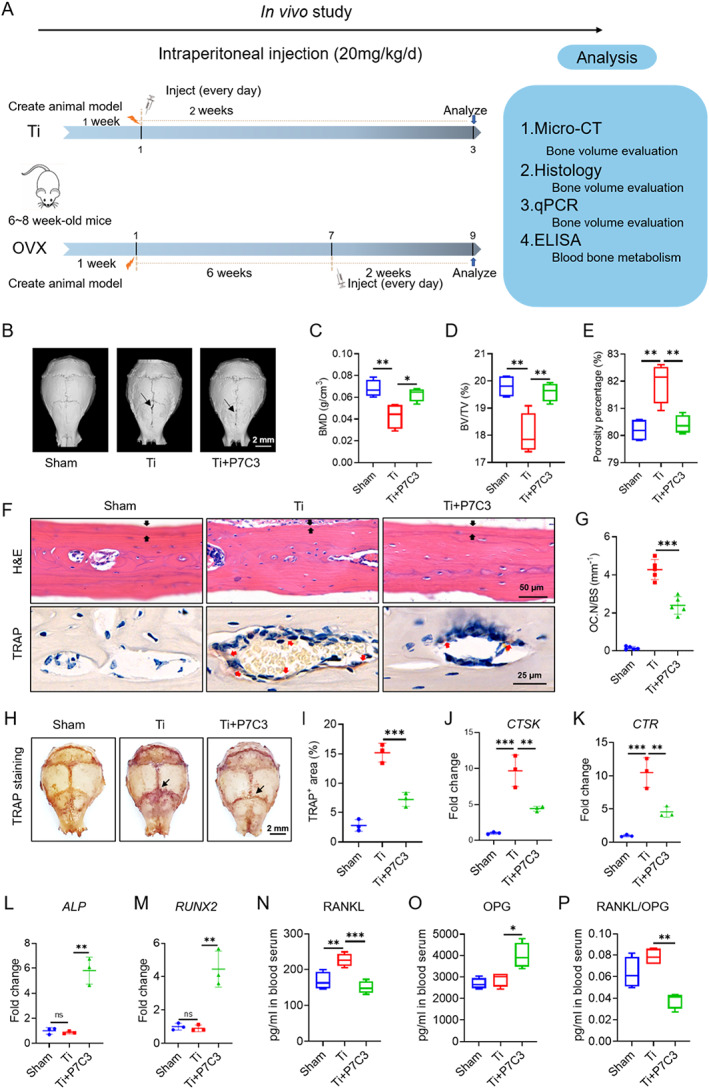
P7C3 suppresses Ti particle‐induced calvarial osteolysis. (*A*) Study design of in vivo animal experiments. (*B*) 3D reconstruction of calvarial bones by μCT. (*C*–*E*) Quantitative analysis of μCT images, including bone mineral density (BMD), bone volume/total volume (BV/TV), and porosity percentage. (*F*) H&E and TRAP staining of calvarial bone tissue, showing the new bone formation and osteoclasts, respectively. (*G*) Relative quantification of TRAP‐positive cells in *F*. (*H*) TRAP staining of osteoclast distribution in whole calvarial bone (*n* = 3). (*I*) Quantification of TRAP‐positive staining area in *H*. (*J*–*M*) qPCR analysis of osteoclast‐specific genes and osteoblast‐specific genes in calvarial bone tissue (*n* = 3). (*N*–*P*) ELISA assay of serum levels of RANKL and OPG, as well as their ratio. Data are presented as mean ± SD; statistical significance was calculated by one‐way ANOVA. *p < 0.05, **p < 0.01, ***p < 0.001. *n* = 5.

To test the potential therapeutic value of P7C3 for bone diseases, we conducted animal experiments using a Ti particle–induced bone destruction model and an OVX‐induced osteoporosis model to explore the potential therapeutic value of P7C3 for bone diseases (Fig. 4*A*). By μCT analysis, we assess the effect of P7C3 on bone destruction in Ti particle–challenged mice. As shown in Fig. 4*B*–*E*, P7C3 treatment significantly inhibited bone erosion, as evidenced by increased bone mineral density and bone volume compared with the Ti particle control group. Hematoxylin and eosin (H&E) staining also confirmed the protective effect of P7C3 on bone tissue and resulted in a marked increase in new bone formation areas compared with the Ti particle control group (Fig. 4*F* and Fig. S2J). Furthermore, TRAP staining of bone slices and whole skull bones showed that P7C3 treatment significantly inhibited the activation of osteoclasts, as evidenced by reduced TRAP‐positive cells (Fig. 4*F*–*I*).

To explore the molecular basis of the in vivo effects of P7C3, we performed real‐time PCR analysis of bone tissues to examine the expression levels of osteoblast‐ and osteoclast‐specific genes. As shown in Fig. 4*J*,*K*, P7C3 treatment significantly inhibited the expression of osteoclast‐specific genes such as *CTSK* and *CTR*, indicating that P7C3 could suppress osteoclast differentiation and activity in vivo. In contrast, the expression levels of osteoblast‐specific genes such as *ALP* and *RUNX2* were significantly upregulated in P7C3‐treated mice, suggesting that P7C3 could promote osteoblast differentiation and bone formation in vivo (Fig. 4*L*,*M*).

In addition, we also examined the serum levels of RANKL and OPG, which were two key factors involved in regulating osteoclast differentiation and activation. As shown in Fig. 4*N*–*P*, P7C3 treatment led to a significant decrease in the serum level of RANKL and an increase in the level of OPG in Ti particle–challenged mice. Furthermore, the ratio of RANKL to OPG was significantly reduced by P7C3 treatment, indicating that P7C3 may regulate bone remodeling by modulating the RANKL/OPG signaling axis. Taken together, these results provide direct evidence that P7C3 can inhibit Ti particle–induced bone erosion, promote new bone formation, and regulate bone remodeling by modulating osteoclast and osteoblast activity.

### P7C3 improved bone remodeling in the OVX‐induced osteoporosis model

To further evaluate the therapeutic potential of P7C3 in bone diseases, we also examined its effects on the development of postmenopausal osteoporosis using an OVX‐induced osteoporosis model. Mice that underwent OVX surgery showed significant weight gain and uterine degeneration compared with the sham‐operated mice, consistent with the characteristics of postmenopausal osteoporosis (Fig. S3A–C).

**Fig. 5:**
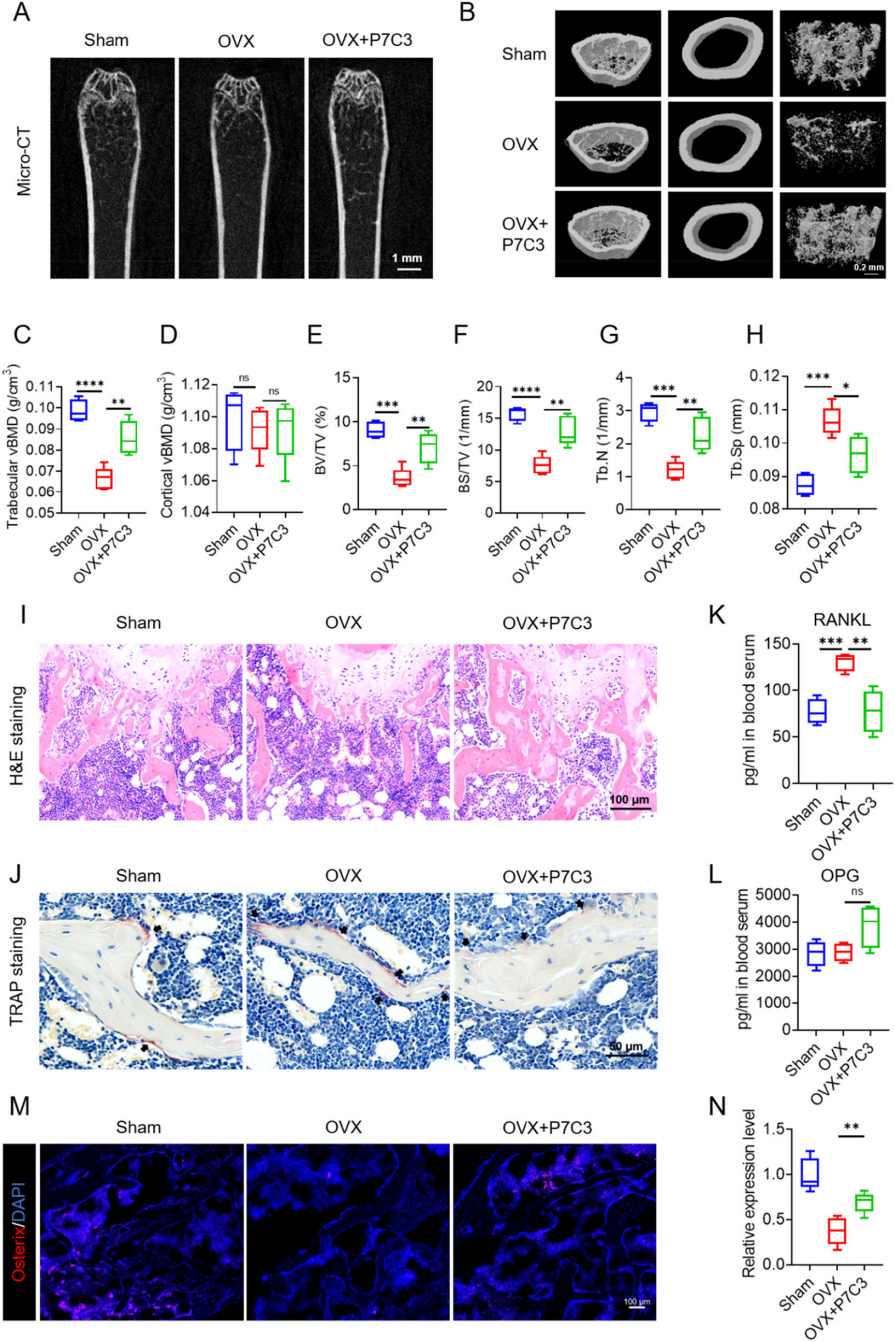
P7C3 improves bone mass in OVX‐induced osteoporosis models. (*A*) 2D cross‐section of mouse femurs by μCT. (*B*) 3D reconstruction of femurs by μCT. (*C*–*H*) parameters, including trabecular bone mineral density, cortical bone mineral density, bone volume/total volume (BV/TV), bone surface area/total volume (BS/TV), trabecular number (Tb.N), and trabecular separation(Tb.Sp). (*I*) H&E staining of bone tissue showing trabecular bone distribution. (*J*) TRAP staining of bone tissue sections showing osteoclasts. (*K*,*L*) ELISA assay of serum levels of RANKL and OPG. (*M*) Immunofluorescence staining of osteoblast‐specific protein osterix expression in bone tissue. (*N*) Relative quantification of osterix protein expression in *M*. Data are presented as mean ± SD; statistical significance was calculated by one‐way ANOVA. *p < 0.05, **p < 0.01, ***p < 0.001, ****p < 0.0001. *n* = 5.

As shown in Fig. 5*A*–*H*, μCT imaging analysis of the femurs from OVX‐induced mice treated with P7C3 revealed a significant increase in bone mass, as evidenced by increased trabecular bone mineral density, trabecular bone volume, bone surface area, and trabecular number, as well as reduced trabecular separation compared with the untreated OVX group. Moreover, histological analysis of bone tissues revealed that P7C3 treatment significantly increased the number of trabeculae and reduced the severity of bone loss in OVX‐induced mice (Fig. 5*I* and Fig. S3D). TRAP staining of bone slices showed that P7C3 treatment significantly inhibited osteoclast activity in OVX‐induced mice which is consistent with the in vitro findings (Fig. 5*J*, and Fig. S3E,F). We also examined the expression levels of RANKL and OPG in the serum of OVX‐induced mice treated with P7C3. P7C3 treatment led to a significant decrease in the level of RANKL and an increase in the level of OPG (Fig. 5*K*,*L*).

Finally, we also examined the expression levels of osteoblast‐specific markers in the bone tissue of OVX‐induced mice treated with P7C3. As shown in Fig. 5*M*,*N*, immunofluorescence staining of bone tissue using an osterix antibody revealed that P7C3 treatment significantly increased the expression of this key osteoblast‐specific marker in bone tissue from OVX‐induced mice. This finding suggests that P7C3 may promote new bone formation and improve bone density in vivo by promoting osteoblast differentiation and activity. Taken together, our results provide direct evidence that P7C3 can effectively attenuate bone loss and improve bone density in OVX‐induced osteoporosis models.

### P7C3 showed excellent biological safety properties

**Fig. 6:**
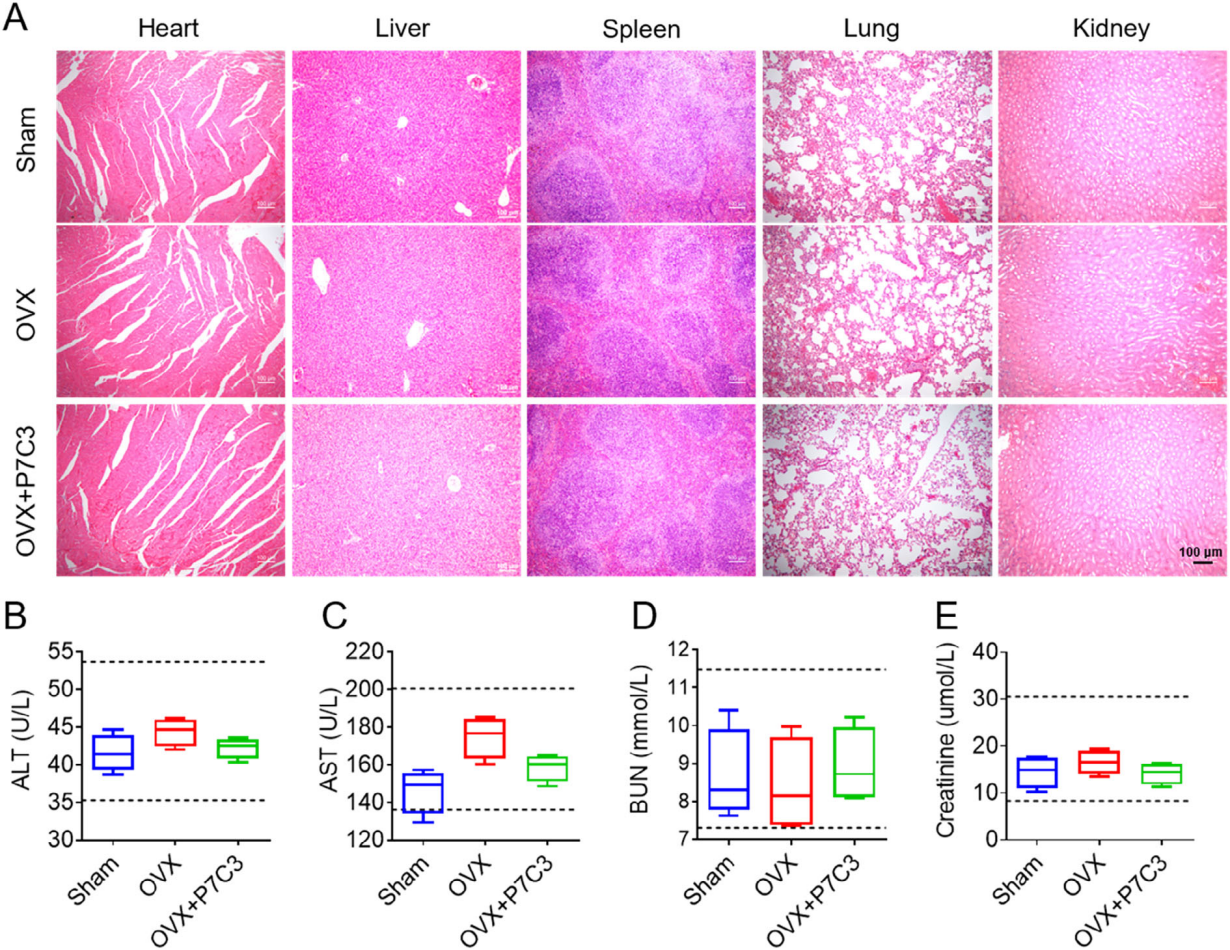
Biological safety of P7C3 in vivo. (*A*) H&E staining of major organs (heart, liver, spleen, lung, and kidney) in mice treated with P7C3 or vehicle control. (*B*–*E*) Serum levels of ALT, AST, BUN, and creatinine in mice treated with P7C3 or vehicle control. Data are presented as mean ± SD; *n* = 5.

To assess the safety and toxicity of P7C3 in vivo, we performed a comprehensive analysis of the major organs in mice treated with P7C3. Compared with the sham‐operated group, P7C3‐treated mice showed no significant signs of toxicity or cellular damage (Fig. 6*A*). Furthermore, we also measured the levels of indicators of liver and kidney function, including ALT, AST, BUN, and creatinine. As shown in Fig. 6*B*–*E*, the levels of ALT and AST in P7C3‐treated mice were within the normal range, indicating that P7C3 treatment did not compromise liver function. Similarly, the levels of BUN and creatinine in P7C3‐treated mice were not significantly different from those in the control group, suggesting that P7C3 did not induce renal damage or affect kidney function. Hence, P7C3 treatment is safe and well‐tolerated in mice, with neglectable toxicity and adverse effects. These findings support the clinical development of P7C3 as a potential therapeutic agent for bone diseases.

## Discussion

**Fig. 7:**
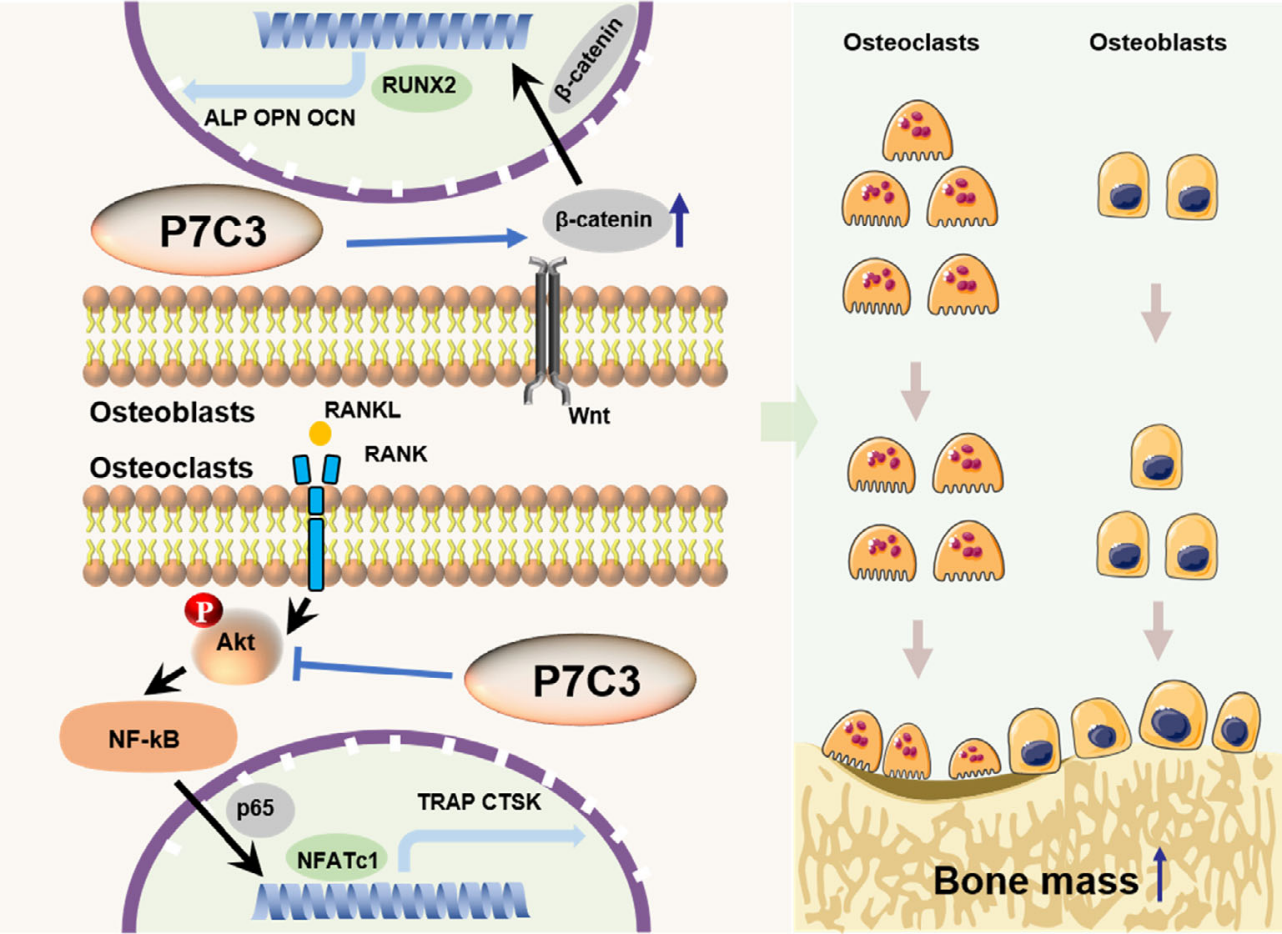
Schematic model outlining the actions of P7C3 on bone. The molecular mechanism of P7C3 in inhibiting osteoclast differentiation and promoting osteoblast differentiation (left) and regulating bone remodeling by suppressing bone resorption and promoting bone formation to increase bone mass (right) have been demonstrated.

In the present study, we investigated the effects of a small molecule compound P7C3 on bone metabolism and its underlying mechanisms. Our findings demonstrate that P7C3 was able to inhibit osteoclast formation while promoting osteoblast differentiation at the same time. (Fig. 7) These effects suggest that P7C3 holds great potential as a therapeutic agent for the treatment of bone diseases, including osteoporosis. Current therapies for osteoporosis employ a range of strategies targeting either bone resorption or bone formation. In addition, most of these drugs carry their safety concerns. Bisphosphonates can cause gastrointestinal side effects, including esophageal irritation, and may increase the risk of osteonecrosis of the jaw.^(^ ^4^, ^39^ ^)^ Denosumab has been linked to an increased risk of fractures risk after discontinuing the drug.^(^ ^1^, ^40^ ^)^ Teriparatide and abaloparatide may increase the risk of osteosarcoma, a type of bone cancer.^(^ ^4^, ^41^ ^)^ Recently, romosozumab, a monoclonal antibody that targets sclerostin has been shown to both reduce bone resorption and increase bone formation. Nevertheless, safety issues such as an increased risk of cardiovascular events have limited its wide use.^(^ ^4^, ^42^ ^)^ A new therapeutic agent for osteoporosis is needed to be developed that can prevent bone resorption and enhance bone formation, without causing undesirable side effects.

Previous studies indicated that P7C3 exerts its broad‐spectrum neuroprotective effects in different species of animals through various cellular mechanisms.^(^ ^26^, ^32^, ^43^, ^44^ ^)^ It has been shown to promote neuronal survival and increase neurite outgrowth, the process by which neurons extend their processes to communicate with other cells.^(^ ^30^, ^32^ ^)^ It has also been demonstrated to suppress microglia‐mediated neuroinflammation by modulating the NF‐κB pathway.^(^ ^45^ ^)^ In this study, we further discovered that P7C3 can inhibit osteoclast differentiation signals by suppressing the upstream phosphorylation of Akt, thereby inhibiting the downstream NF‐κB signal. The inhibition of the Akt‐NF‐κB‐NFATc1 signaling pathway by P7C3 provides a clear molecular mechanism for its anti‐osteoclastic activity. This pathway has been reported to be responsible for osteoclast differentiation and is also closely related to pathologies such as osteoarthritis.^(^ ^46^ ^)^ Previous studies have shown that inhibition of Akt‐NF‐κB‐NFATc1 pathway components can reduce bone resorption and prevent bone loss in animal models.^(^ ^47^, ^48^ ^)^ Our results support this finding, and demonstrate the value of targeting this pathway in developing novel therapies for osteoporosis and other bone‐related disorders.

The promotion of osteoblast differentiation by P7C3 is also a crucial finding. The Wnt/β‐catenin signaling pathway has been regarded as an important regulator of osteoblastogenesis and bone formation.^(^ ^49^, ^50^, ^51^ ^)^ We provide informative evidence that P7C3 can activate this pathway and promote osteoblast differentiation. This is a promising finding, as a decline in osteoblast differentiation and bone formation is a hallmark of bone diseases such as osteoporosis and osteogenesis imperfecta.^(^ ^52^ ^)^

More importantly, P7C3 has shown remarkable safety and biocompatibility in animal models, with neglectable apparent adverse effects during extensive investigations for neurodegenerative disorders in previous and current studies.^(^ ^23^, ^26^, ^31^ ^)^ These properties make P7C3 an attractive candidate for the development of new therapies for the treatment of osteoporosis.

In conclusion, our study has explored the mechanism and extended the use of P7C3 in bone disease treatment. The findings of this study offer a promising outlook for the therapeutic potential of P7C3 in the treatment of bone‐related disorders such as osteoporosis, which is a prevalent medical condition affecting millions of people worldwide. Furthermore, given its neuroprotective properties, P7C3 has potential therapeutic benefits for age‐related neurodegenerative diseases.^(^ ^23^, ^26^, ^53^ ^)^ As both osteoporosis and neurodegenerative diseases are age‐related diseases,^(^ ^54^ ^)^ the potential for a single drug to treat both conditions could have significant clinical implications. Thus, P7C3 warrants further investigation to establish its safety, efficacy, and optimal therapeutic dosing in human clinical trials. The potential for P7C3 to be used in combination with existing treatments for osteoporosis and other bone diseases also merits further exploration.

## Author Contributions

**Bo Tian:** Conceptualization; data curation; formal analysis; investigation; methodology; writing – original draft. **Jinyu Bai:** Conceptualization; data curation; formal analysis; funding acquisition; investigation; methodology. **Lei Sheng:** Data curation; formal analysis; investigation; methodology. **Hao Chen:** Investigation. **Wenju Chang:** Formal analysis. **Yue Zhang:** Investigation; methodology. **Chenlu Yao:** Methodology; writing – review and editing. **Chenmeng Zhou:** Investigation; methodology. **Xiaoyu Wang:** Formal analysis. **Huajian Shan:** Methodology; project administration. **Qirong Dong:** Conceptualization; funding acquisition; methodology; supervision. **Chao Wang:** Conceptualization; funding acquisition; supervision; writing – original draft; writing – review and editing. **Xiaozhong Zhou:** Conceptualization; funding acquisition; methodology; supervision; writing – review and editing.

### Peer Review

The peer review history for this article is available at https://www.webofscience.com/api/gateway/wos/peer-review/10.1002/jbm4.10811.

## Disclosures

All authors report no conflicts of interest.

## Supporting information


**Fig. S1.** Effects of P7C3 on cell proliferation and viability. (A, B) MTT assay showing the viability of osteoclast precursor cells treated with P7C3 for 24 or 48 h. (C, D) MTT assay showing the viability of osteoblast precursor cells treated with P7C3 for 24 or 48 h. Data are presented as mean ± SD; *n* = 6.
**Fig. S2.** Quantitative analysis. (A) Quantitative analysis of Fig. 1K. (B–D) Relative protein expression quantification of Fig. 2A. (E) Relative protein quantification analysis of Fig. 2F. (F–H) Relative protein expression quantification of Fig. 2G. (I) Relative protein quantification analysis of Fig. 3M. (J) Morphological quantification analysis of H&E‐stained new bone formation area in Fig. 4F (*n* = 5). Data are presented as mean ± SD; Statistical significance was calculated by one‐way ANOVA and Student's *t* test. *p < 0.05, **p < 0.01, ***p < 0.001, ****p < 0.0001. *n* = 3.
**Fig. S3.** Confirmation of OVX‐induced osteoporotic animal model and quantitative analysis of bone morphology. (A) Body weight changes of mice after OVX surgery. (B) Uterine atrophy at 8 weeks post‐surgery. (C) Quantification of uterine weight in (B). (D) Morphometric measurement analysis of bone in Fig. 5I. (E) and (F) Relative quantification analysis of TRAP‐stained osteoclast formation on bone surface in Fig. 5J. BV/TV, bone volume per tissue volume; OC.S/BS, osteoclast surface area per bone surface; OC.N/BS, osteoclast number per bone surface. Data are presented as mean ± SD; Statistical significance was calculated by Student's t test and one‐way ANOVA. *p < 0.05, **p < 0.01, ***p < 0.001, ****p < 0.0001. *n* = 5.
**Fig. S4.** Original data of western blots in the paper. (A) Original western blot image for Fig. 1G. (B) Original western blot image for Fig. 2A. (C) Original western blot image for Fig. 2B. (D) Original western blot image for Fig. 2F. (E) Original western blot image for Fig. 2G. (F) Original western blot image for Fig. 3M.
**Table S1.** Specific Primer Sequences for qPCR Analysis

## Data Availability

The data that support the findings of this study are available from the corresponding author upon reasonable request.
