# Supplementary material for: P7C3 Ameliorates Bone Loss by Inhibiting Osteoclast Differentiation and Promoting Osteogenesis
Source: JBMR Plus. 2023 Sep 6;7(12):e10811. doi: 10.1002/jbm4.10811 (PMC10731119; doi:10.1002/jbm4.10811)
Supplement: Supplementary file 1 — Fig. S1. Effects of P7C3 on cell proliferation and viability. (A, B) MTT assay showing the viability of osteoclast precursor cells treated with P7C3 for 24 or 48 h. (C, D) MTT assay showing the viability of osteoblast precursor cells treated with P7C3 for 24 or 48 h. Data are presented as mean ± SD; n = 6. Fig. S2. Quantitative analysis. (A) Quantitative analysis of Fig. 1K. (B–D) Relative protein expression quantification of Fig. 2A. (E) Relative protein quantification analysis of Fig. 2F. (F–H) Relative protein expression quantification of Fig. 2G. (I) Relative protein quantification analysis of Fig. 3M. (J) Morphological quantification analysis of H&E‐stained new bone formation area in Fig. 4F (n = 5). Data are presented as mean ± SD; Statistical significance was calculated by one‐way ANOVA and Student's t test. *p < 0.05, **p < 0.01, ***p < 0.001, ****p < 0.0001. n = 3. Fig. S3. Confirmation of OVX‐induced osteoporotic animal model and quantitative analysis of bone morphology. (A) Body weight changes of mice after OVX surgery. (B) Uterine atrophy at 8 weeks post‐surgery. (C) Quantification of uterine weight in (B). (D) Morphometric measurement analysis of bone in Fig. 5I. (E) and (F) Relative quantification analysis of TRAP‐stained osteoclast formation on bone surface in Fig. 5J. BV/TV, bone volume per tissue volume; OC.S/BS, osteoclast surface area per bone surface; OC.N/BS, osteoclast number per bone surface. Data are presented as mean ± SD; Statistical significance was calculated by Student's t test and one‐way ANOVA. *p < 0.05, **p < 0.01, ***p < 0.001, ****p < 0.0001. n = 5. Fig. S4. Original data of western blots in the paper. (A) Original western blot image for Fig. 1G. (B) Original western blot image for Fig. 2A. (C) Original western blot image for Fig. 2B. (D) Original western blot image for Fig. 2F. (E) Original western blot image for Fig. 2G. (F) Original western blot image for Fig. 3M. Table S1. Specific Primer Sequences for qPCR Analysis [file JBM4-7-e10811-s001.docx]

**Supplementary Materials for**

**P7C3 Ameliorates Bone Loss by Inhibiting Osteoclast Differentiation and Promoting Osteogenesis**

Bo Tian^1,2§^, Jinyu Bai^1§^, Lei Sheng ^1§^, Hao Chen^1^, Wenju Chang^1^, Yue Zhang^2^, Chenlu Yao^2^, Chenmeng Zhou^2^, Xiaoyu Wang^2^, Huajian Shan^1^, Qirong Dong^1∗^, Chao Wang^2∗^, and Xiaozhong Zhou^1∗^

^1^Department of Orthopedics, The Second Affiliated Hospital of Soochow University, Suzhou, Jiangsu 215004, China

^2^Laboratory for Biomaterial and ImmunoEngineering, Institute of Functional Nano & SoftMaterials (FUNSOM), Soochow University, Suzhou, Jiangsu 215123, China

^§^ These authors contributed equally to this work.

^∗^ Corresponding to: Qirong Dong, E-mail: [dongqirong@suda.edu.cn](mailto:dongqirong@suda.edu.cn); Chao Wang, E-mail: [cwang@suda.edu.cn](mailto:cwang@suda.edu.cn); Xiaozhong Zhou, E-mail: [zhouxz@suda.edu.cn](mailto:zhouxz@suda.edu.cn)

Address: 1055 San'Xiang Road

Department of Orthopedics, The Second Affiliated Hospital of Soochow University, Suzhou, Jiangsu 215004, China

199 Ren'Ai Road

Laboratory for Biomaterial and ImmunoEngineering, Institute of Functional Nano & SoftMaterials (FUNSOM), Soochow University, Suzhou, Jiangsu 215123, China

**Supplementary Figures**


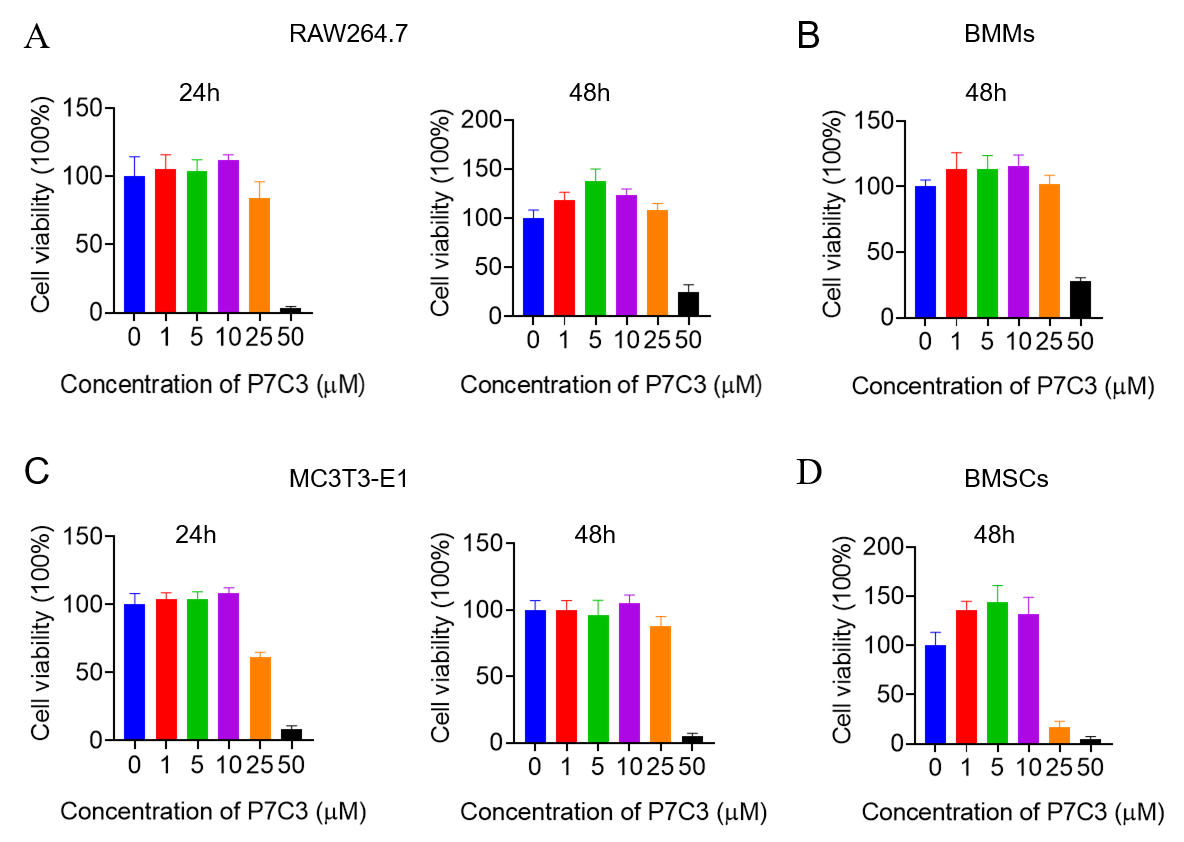


**Figure S1**. Effects of P7C3 on cell proliferation and viability. (A-B) MTT assay showing the viability of osteoclast precursor cells treated with P7C3 for 24 or 48 hours. (C-D) MTT assay showing the viability of osteoblast precursor cells treated with P7C3 for 24 or 48 hours. Data are presented as mean ± SD; n=6.


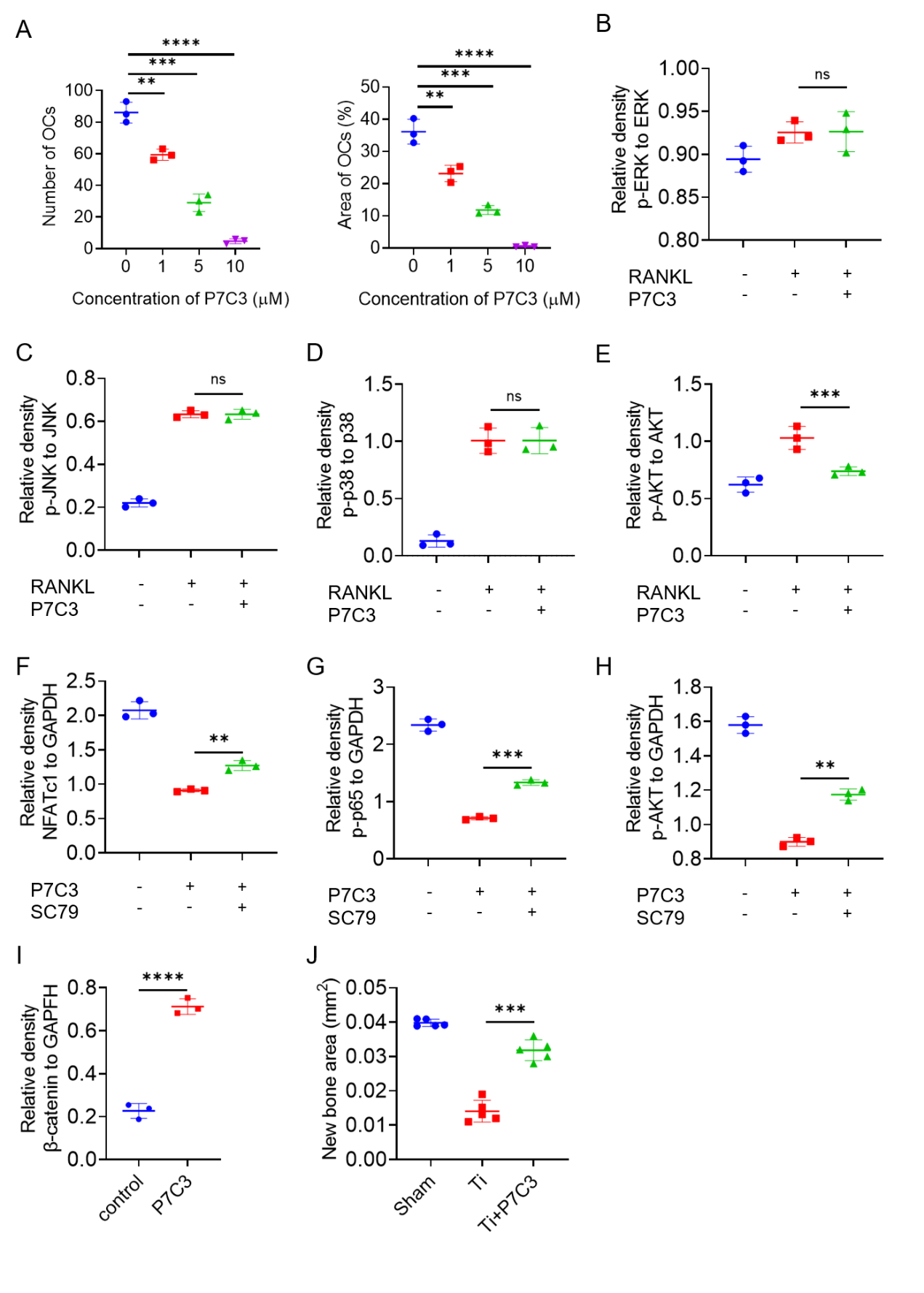


**Figure S2.** Quantitative analysis. (A) Quantitative analysis of Figure 1K. (B-D) Relative protein expression quantification of Figure 2A. (E) Relative protein quantification analysis of Figure 2F. (F-H) Relative protein expression quantification of Figure 2G. (I) Relative protein quantification analysis of Figure 3M. (J) Morphological quantification analysis of H&E-stained new bone formation area in Figure 4F (n=5). Data are presented as mean ± SD; Statistical significance was calculated by one-way ANOVA and Student’s t test. *p<0.05, **p<0.01, ***p<0.001, ****p<0.0001. n=3.


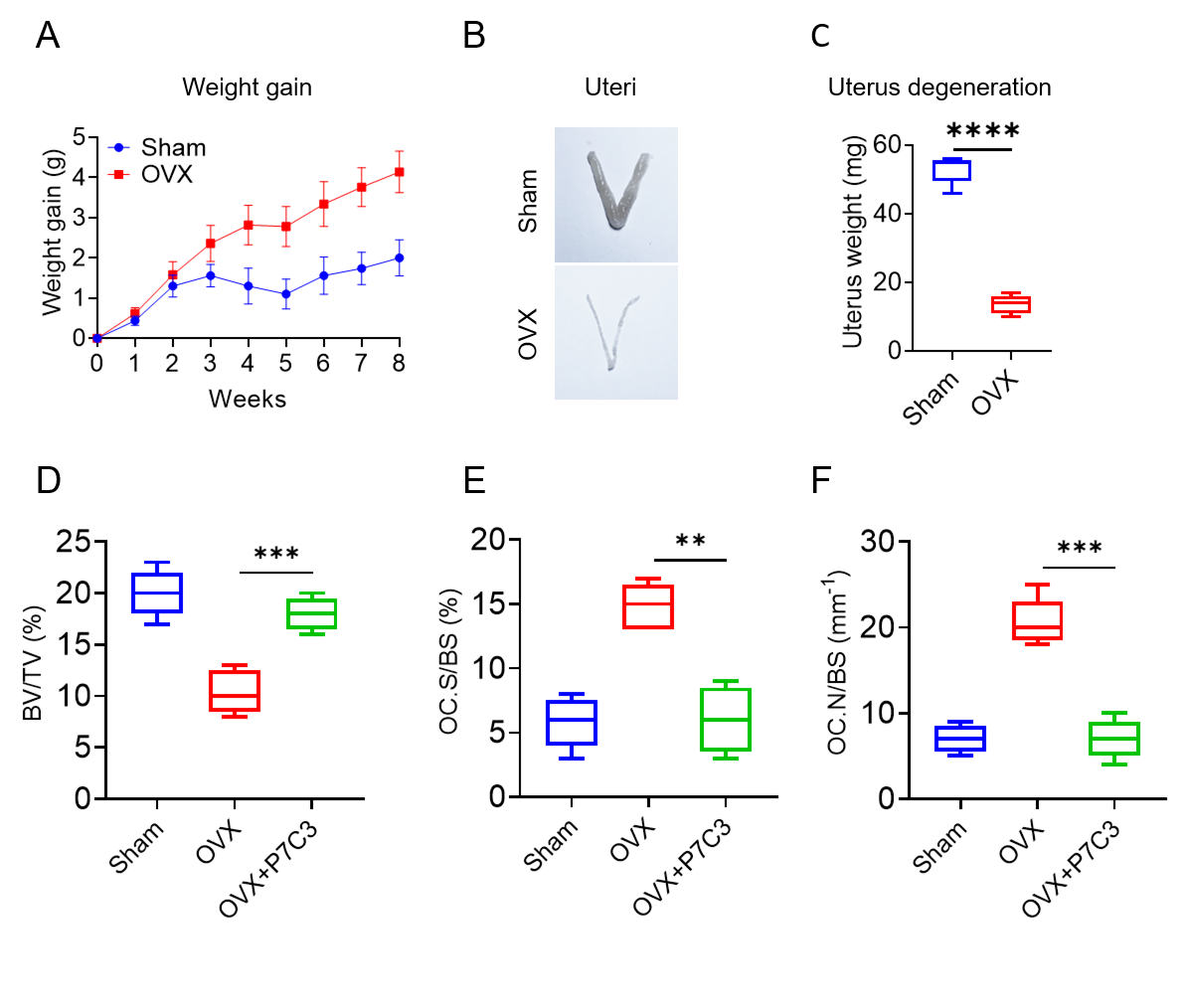


**Figure S3**. Confirmation of OVX-induced osteoporotic animal model and quantitative analysis of bone morphology. (A) Body weight changes of mice after OVX surgery. (B) Uterine atrophy at 8 weeks post-surgery. (C) Quantification of uterine weight in (B). (D) Morphometric measurement analysis of bone in Figure 5I. (E) and (F) Relative quantification analysis of TRAP-stained osteoclast formation on bone surface in Figure 5J. BV/TV, bone volume per tissue volume; OC.S/BS, osteoclast surface area per bone surface; OC.N/BS, osteoclast number per bone surface. Data are presented as mean ± SD; Statistical significance was calculated by Student’s t test and one-way ANOVA. *p<0.05, **p<0.01, ***p<0.001, ****p<0.0001. n=5.


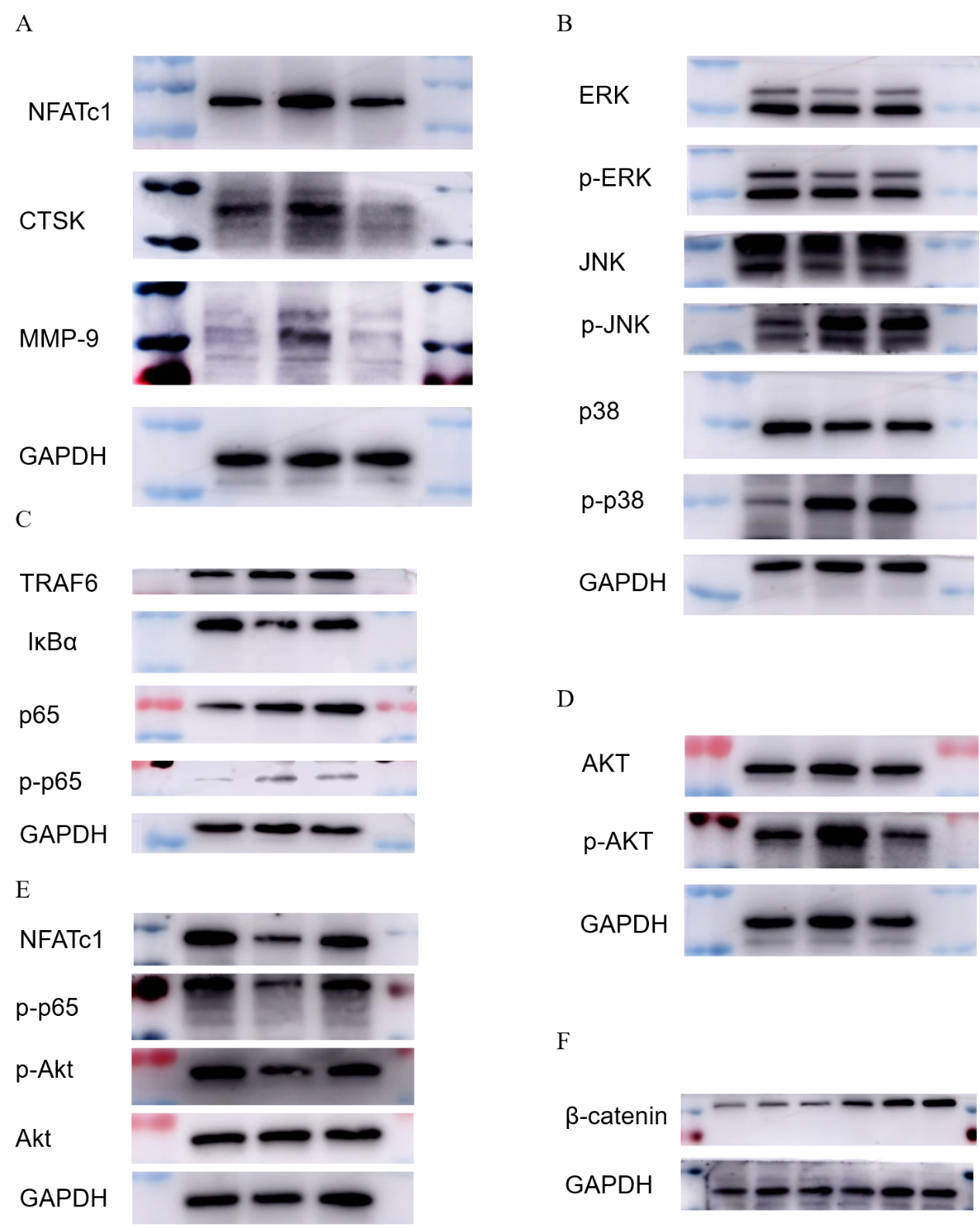


**Figure S4**. Original data of western blots in the paper. (A) Original western blot image for Figure 1G. (B) Original western blot image for Figure 2A. (C) Original western blot image for Figure 2B. (D) Original western blot image for Figure 2F. (E) Original western blot image for Figure 2G. (F) Original western blot image for Figure 3M.

**Supplementary Table**


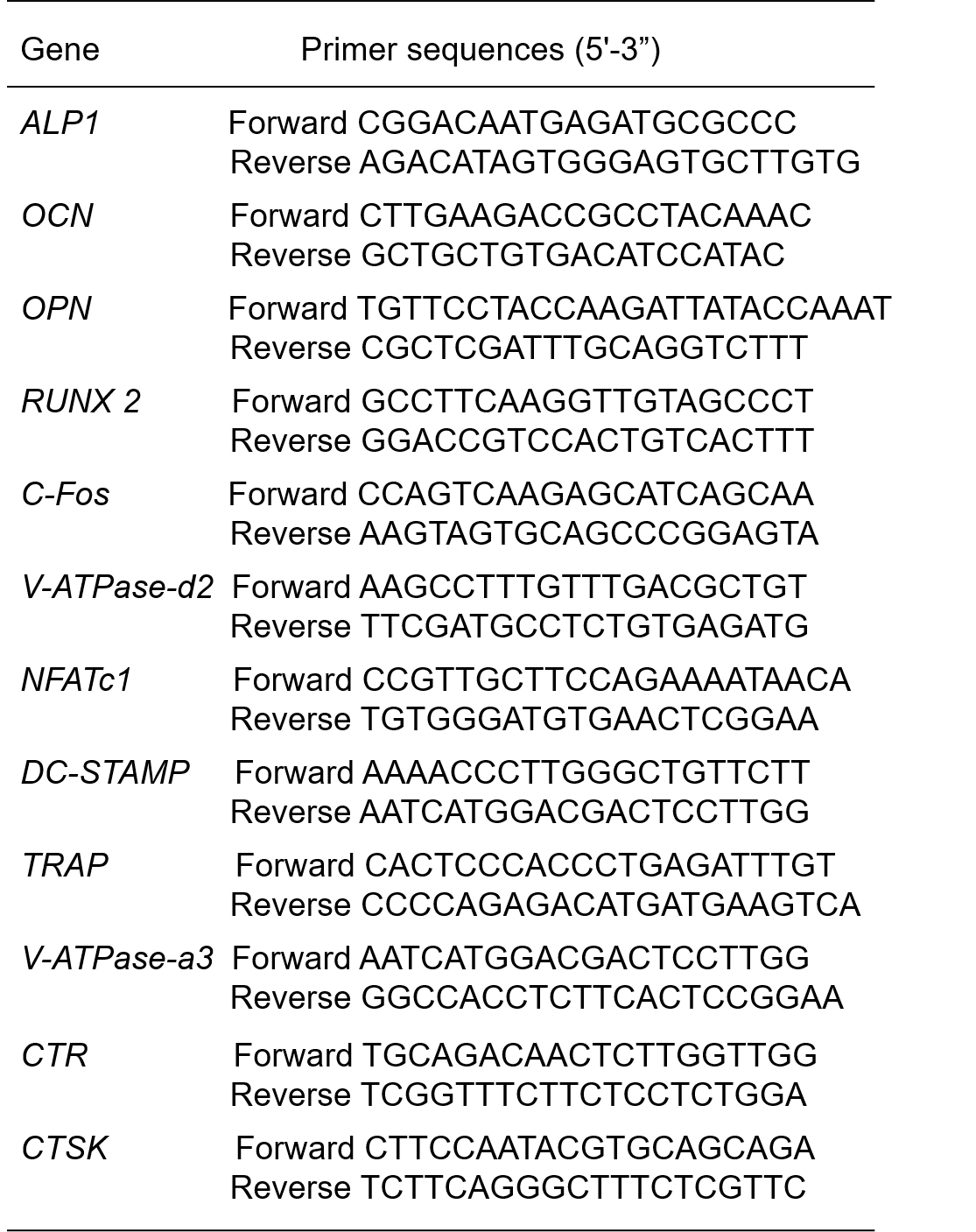


**Table S1.** Specific primer sequences for qPCR analysis.
